# Expression of K1 Toxin Derivatives in *Saccharomyces cerevisiae* Mimics Treatment with Exogenous Toxin and Provides a Useful Tool for Elucidating K1 Mechanisms of Action and Immunity

**DOI:** 10.3390/toxins9110345

**Published:** 2017-10-27

**Authors:** Stefanie Gier, Manfred J. Schmitt, Frank Breinig

**Affiliations:** 1 Center for Human and Molecular Biology (ZHMB), Saarland University, D-66123 Saarbrücken, Germany; stefanie.gier@uni-saarland.de (S.G.); mjs@microbiol.uni-sb.de (M.J.S.); 2 Molecular and Cell Biology, Campus A1.5, Saarland University, D-66123 Saarbrücken, Germany

**Keywords:** yeast viral toxin, K1, *Saccharomyces cerevisiae*

## Abstract

Killer toxin K1 is a heterodimeric protein toxin secreted by *Saccharomyces cerevisiae* strains infected with the M1 double-stranded RNA ‘killer’ virus. After binding to a primary receptor at the level of the cell wall, K1 interacts with its secondary plasma membrane receptor Kre1p, eventually leading to an ionophoric disruption of membrane function. Although it has been under investigation for decades, neither the particular mechanisms leading to toxicity nor those leading to immunity have been elucidated. In this study, we constructed derivatives of the K1α subunit and expressed them in sensitive yeast cells. We show that these derivatives are able to mimic the action of externally applied K1 toxin in terms of growth inhibition and pore formation within the membrane, leading to a suicidal phenotype that could be abolished by co-expression of the toxin precursor, confirming a mechanistic similarity of external and internal toxin action. The derivatives were successfully used to investigate a null mutant completely resistant to externally applied toxin. They provide a valuable tool for the identification of so far unknown gene products involved in K1 toxin action and/or immunity.

## 1. Introduction

The killer phenomenon in yeast was first described in 1963, and has been shown since then to be widespread amongst different yeast genera [1,2]. Based on the secretion of proteinous toxins—the so-called killer toxins—killer strains possess the ability to kill sensitive yeast cells without direct interaction; killer cells are simultaneously immune to their own toxin. Among these specific yeast toxins, the killer toxins of the baker’s yeast *Saccharomyces cerevisiae*, K1 and K28, belong to the best-studied proteins [3,4]. The killer phenomenon relies on two different dsRNA viruses (named L-A and M) which are persistently present as virus-like particles (VLPs) in the cytoplasm of the infected yeast cell [5]. L-A mycoviruses are self-replicating so-called helper viruses, encoding the capsid protein (Gag) and a RNA-dependent RNA polymerase that is expressed as a Gag-Pol fusion protein (Gag/Pol). In contrast, the satellite M virus contains solely the genetic information for the respective killer toxin and exploits L-A Gag and Gag/Pol. Consequently, the presence of L-A viruses is crucial for the maintenance and replication of both mycoviruses while the M virus is responsible for the development of a killer phenotype and the confirmation of self-immunity [4].

Within the killer cell, K1 and K28 are initially synthesized as precursor proteins (preprotoxin, pptox), which are processed within the secretory pathway and eventually secreted as functional heterodimeric AB toxins. Behind an N-terminal signal sequence responsible for ER import and a pro-region with so far unknown function, both toxins contain an α and β subunit, which are separated by a possibly N-glycosylated γ subunit. Whereas the β subunit is essential for toxin binding to the potential target cell, the α subunit has been shown to mediate the toxic effect. Within the ER, the signal sequence is cleaved off and α and β are connected via one or more disulphide bond/s. Subsequently, the toxin precursor is transported to the Golgi and undergoes further processing. In a late-Golgi compartment, the pro-region and the γ sequence are removed by the endopeptidase Kex2p and the C termini of both subunits are trimmed by the carboxypeptidase Kex1p finally resulting in a mature α/β toxin molecule [6,7].

Although K1 and K28 have a similar structural composition, their modes of action are completely different. After binding to a cell wall mannoprotein and its membrane receptor Erd2p, K28 enters the cell via endocytosis and by parasitizing the retrograde secretory pathway, finally reaching the nucleus, where it provokes both blockage of DNA synthesis and cell cycle arrest [8,9]. In contrast, K1 acts as an ionophore, initially binding to β-1,6 glucans in the yeast cell wall as primary receptor [10]. The toxin is then transported to the plasma membrane in an energy-dependent process where it interacts with its membrane receptor, GPI-anchored Kre1p, subsequently leading to the disruption of membrane integrity by forming cation-selective channels and finally culminating in cell death [11]. 

Despite having been under investigation for several decades, the exact molecular mechanisms underlying K1 toxin action and immunity still remain to be elucidated. In particular, analysis of several yeast deletion mutants is hampered by the fact that the respective gene products have pleiotropic effects and are involved in, or at least have a significant influence on, presence and/or amount of the toxin’s cell wall receptor β-1,6-glucan. As a consequence, the cells become resistant to externally applied toxin. In this study, we constructed several K1 derivatives for intracellular expression in *S. cerevisiae* to circumvent this issue. We show that these derivatives mimic the action of externally applied toxin, and use them as a tool to prove that the K1 membrane receptor Kre1p is neither involved in toxin action nor immunity.

## 2. Results

To shed some light on the mechanisms of K1 immunity or its lethal effect, Pagé et al. performed a genome-wide screening with all available deletion mutants of *S. cerevisiae* using externally applied K1 toxin [12]. However, this “classical” approach is hampered by the fact that many mutants show defects in their cell wall composition, including the K1 receptor β-1,6-glucan, resulting in a resistance to external toxin. Thus, there is a need for novel approaches and tools that allow screening of individual deletion mutants without external application of the toxin.

### 2.1. Expression of K1α Derivatives Mimics Natural Toxin Action 

**Figure 1:**
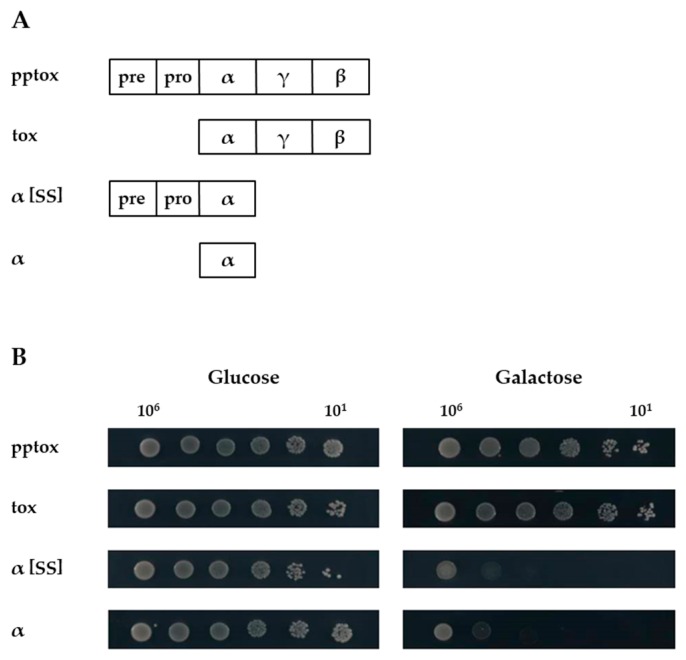
(**A**) **K1 derivatives used in this study**. Schematic drawing of the constructed K1 derivatives. The toxin precursor (pptox) containing the ER import signal (pre) and the pro-region (pro) and a toxin variant lacking the prepro-region (tox) were used as controls. K1 derivatives contained only the toxic α subunit either with (α[SS]) or without prepro-region (α). (**B**) **Expression of K1*α* derivatives leads to cell death**. Yeast strain *S. cerevisiae* BY4742 was transformed with the indicated K1 derivatives and 10^6^ to 10^1^ cells were spotted onto either glucose- (left, non-induced condition) or galactose- (right, induced condition) containing agar plates. Growth was analyzed after 3 days at 30 °C. One representative experiment is shown (*n* = 5).

To fill this gap, we constructed four different K1 derivatives, and expressed them in the sensitive yeast strain BY4742 under transcriptional control of the inducible *GAL1* promoter (Figure 1A). As previous analyses using site-directed mutagenesis indicated that the α subunit mediates the toxic effect of K1 [13], we focused on expression of this subunit either with or without prepro-region (α[SS] or α, respectively). Additionally, we used whole pptox and tox (i.e., pptox lacking the prepro-region) as controls.

All transformants grew equally well under non-induced conditions using glucose as carbon source (Figure 1B, left). In contrast, the expression of both K1α derivatives yielded a strong suicidal phenotype indicated by a clear decrease in cell growth on galactose, whereas K1 pptox- and tox-expressing yeast cells showed no growth impairment even under induced conditions (Figure 1B, right). Interestingly, no significant difference between α and α[SS] could be observed, pointing clearly to a lethal intracellular activity of α without the need of being imported into the secretory pathway.

**Figure 2:**
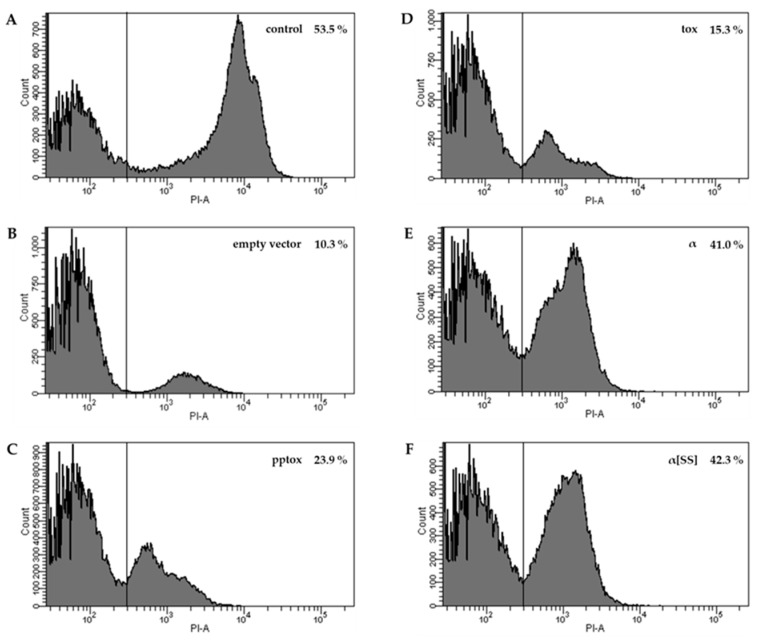
**K1 derivatives induce pore formation in the membrane of a sensitive yeast strain**. Transformants of *S. cerevisiae* BY4742 containing the indicated K1 derivative were grown in galactose-containing medium for 24 h at 30 °C to induce expression and stained with 1 µg/mL PI. For each experiment, 50,000 events were counted. Untransformed yeast cells incubated with K1 toxin (**A**) and cells transformed with an empty vector (**B**) were used as positive and negative controls, respectively. Yeast cells containing whole K1 preprotoxin (pptox, **C**), K1 preprotoxin lacking the prepro-region (tox, **D**), K1α subunit without prepro-region (α, **E**) or K1α subunit with prepro-region (α[SS], **F**). The respective percentage of PI-positive yeast cells is shown in the upper right.

As it is well known that externally applied K1 causes pores within the plasma membrane of sensitive cells, we then investigated a potential pore-forming effect of the different toxin derivatives using propidium iodide (PI) staining and subsequent flow cytometry (Figure 2). As expected, external K1 application led to a clear formation of pores and a significant number of PI-positive cells (Figure 2A). Remarkably, although no growth defect was visible in the serial dilution assay, expression of K1-pptox caused a positive PI staining (23.9%) in comparison to control cells transformed with an empty vector (Figure 2B, 10.3%), also implying some pore formation (Figure 2C).

This observation is in agreement with published data describing an incomplete mediation of immunity by the toxin precursor itself after expression from an episomal plasmid [14]. Likewise, the intracellular expression of the K1-tox derivative caused some PI-positive cells (15.3%, Figure 2D). Nevertheless, the data clearly confirm that expression of K1-α and K1-α[SS] (Figure 2E,F) yielded a disruption of the plasma membrane confirming a pore-forming capacity of the toxin derivatives. Again, there was no difference between the α derivatives with K1-α resulting in 41.0% and K1-α[SS] inducing 42.3% PI-positive cells, respectively. 

### 2.2. Co-Expression of K1 Toxin Precursor Abolishes the Suicidal Phenotype of K1α Derivatives

Having shown that the lethal effect of the K1α derivatives is based on the formation of pores within the plasma membrane, we next asked if the mechanisms of pore formation from outside (externally applied toxin) and inside (expression of K1α derivatives) are mechanistically comparable or different. It is well known from the literature that pptox expression confers immunity to external toxin [15]. Thus, a rather simple way to answer this question is to test if co-expression of pptox would abolish the observed growth defect caused by the K1α derivatives.

**Figure 3:**
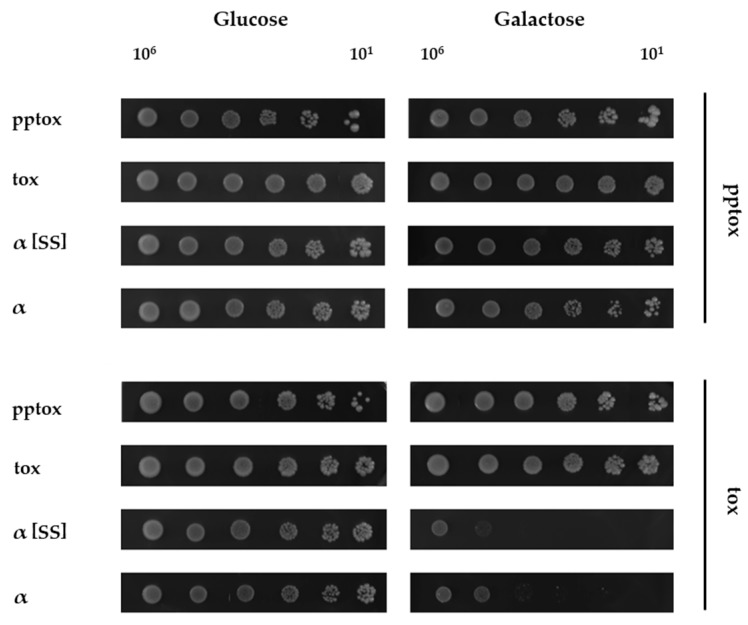
**K1 pptox abolishes the suicidal phenotype of K1*α* derivatives**. *S. cerevisiae* BY4742 containing K1 pptox or K1 tox was co-transformed with the indicated K1 derivatives (depicted on the left) and 10^6^ to 10^1^ cells were spotted onto either glucose- (**left**, non-induced condition) or galactose- (**right**, induced condition) containing agar plates. Growth was analyzed after 3 days at 30 °C. One representative experiment is shown (*n* = 5).

To do so, either K1-pptox or K1-tox were subcloned into vector pYX242 under transcriptional control of the constitutive *TPI1* promoter. Then, the K1 derivatives shown in Figure 1 were co-transformed with either pptox or tox and growth was checked again using a serial dilution assay (Figure 3).

When cultivated under non-induced conditions with glucose as carbon source, all co-transformed yeast cells grew equally well, and no growth defect was detected (Figure 3, left). However, under induced conditions, co-expression of pptox was able to abolish the suicidal phenotype of both K1*α* constructs completely, strongly indicating that pptox confers functional immunity in both cases (Figure 3, right). This observation clearly suggests that the lethal effects of external toxin and the K1*α* derivatives constructed in this study are mechanistically comparable. Interestingly, co-expression of pYX242-tox influenced neither K1α nor K1α[SS] lethality, demonstrating that functional immunity requires the import of the precursor into the secretory pathway. In agreement with this, tox-expressing cells were not immune to external toxin (not shown).

### 2.3. Kre1p Is Involved in Neither K1 Lethality nor Immunity

Previously, we have identified Kre1p as K1 plasma membrane receptor and, consequently, cells with a null mutation (*Δkre1*) show a complete resistance to externally applied K1 toxin at the membrane level [11,16]. To make things worse, Kre1p is itself involved in the synthesis of the K1 cell wall receptor β-1,6-glucan and, thus, the respective mutant is not susceptible to externally applied toxin at all [16,17]. Therefore, until now, it has remained unclear whether there is an additional function of Kre1p in the mechanisms leading to K1 lethality or, in particular, immunity downstream of K1 binding at the membrane.

**Figure 4:**
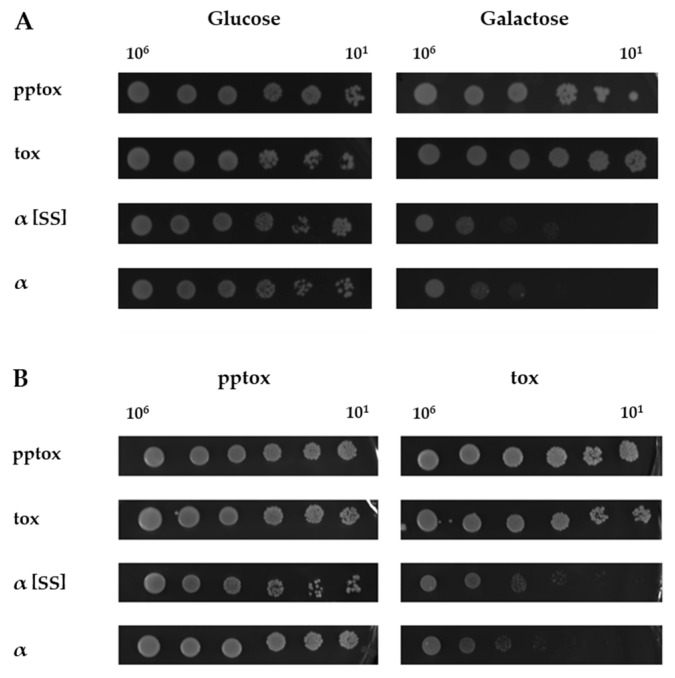
**Kre1p is involved in neither K1 toxicity nor immunity.** (**A**) **Expression of K1*α* derivatives leads to cell death in a *Δkre1* null mutant**. Yeast strain *S. cerevisiae* BY4742 *Δkre1* was transformed with the indicated K1 derivatives and 10^6^ to 10^1^ cells were spotted onto either glucose- (**left**, non-induced condition) or galactose- (**right**, induced condition) containing agar plates. Growth was analyzed after 3 days at 30 °C. One representative experiment is shown (*n* = 5). (**B**) **K1 pptox abolishes the suicidal phenotype of K1*α* derivatives in a *Δkre1* null mutant**. *S. cerevisiae* BY4742 containing K1 pptox or K1 tox was co-transformed with the indicated K1 derivatives (depicted on the left) and 10^6^ to 10^1^ cells were spotted onto either glucose- (**left**, non-induced condition) or galactose- (**right**, induced condition) containing agar plates. Growth was analyzed after 3 days at 30 °C. One representative experiment is shown (*n* = 5).

As the K1α derivatives constructed in this study obviously mimic the action of external toxin but are, unlike external toxin, not dependent on binding to cell wall and membrane receptors, we used them as a tool to investigate the role of Kre1p in more detail. For this purpose, a *Δkre1* null mutant was transformed with the K1 derivatives described above and growth was checked using a serial dilution assay (Figure 4). As seen in the corresponding wild type (see Figure 1B), growth of the transformed null mutant was also unaffected on glucose, but was heavily impaired after expression of the K1α derivatives on galactose, independent of the presence of an ER-import signal (Figure 4A). Likewise, coexpression of K1-pptox abolished the suicidal phenotype completely, whereas K1-tox was not able to confer immunity (Figure 4B). These data clearly exclude a role of Kre1p in K1 lethality or immunity, and prove the usefulness of the constructed K1α derivatives as a novel tool in K1 research.

## 3. Discussion

In contrast to bacterial toxins, which selectively attack eukaryotic targets, killer yeast cells are, in principle, susceptible to their own toxins, making functional immunity crucial for cell survival. As we have shown previously, K28 immunity is based on an intrinsic mechanism in which the toxin precursor interacts with re-endocytosed K28 in the cytoplasm of the killer cell [18]. Some years ago, mechanisms for K1 lethality and immunity relying on an interaction of K1 or K1 pptox with the potassium channel Tok1p were proposed [19,20], which, meanwhile, have been repeatedly shown to be of little significance in vivo [11,12,21]. Thus, the molecular mechanisms leading to both K1 immunity and toxicity still remain to be elucidated. 

The classical screening approach of deletion mutants by externally applying K1 toxin is severely hampered by the fact that several mutations lead pleiotropically to changes in cell wall composition. This renders the particular mutants resistant, as the toxin is no longer able to bind to its primary β-1,6-glucan receptor, and possible interactions of K1 with intracellular proteins cannot be studied. In this study, in order to overcome this obstacle, we constructed different K1α derivatives and demonstrate their ability to mimic the treatment with exogenous toxin with regard to lethality and pore-forming capacity after expression in a sensitive yeast strain. Interestingly, no significant differences between α and α[SS] could be detected, implying that neither import of α into the secretory pathway nor the presence of the β subunit are a prerequisite for its lethal action. This observation adds a fascinating new facet to K1 biology, as an effect ‘from inside’ has never been reported before, providing a means for novel insights into the molecular events that eventually lead to lethality in future experiments.

Most importantly, co-expression of pptox was able to confer immunity against both α and α[SS], clearly confirming that the lethal effects of externally applied K1 toxin and the constructed α derivatives are mechanistically similar. In these experiments, co-expression of the precursor lacking its signal sequence (tox) had no protective effect, suggesting that functional immunity requires import of the precursor into the secretory pathway and, obviously, is fundamentally different to the mechanism underlying K28 immunity [18]. It should be particularly noted that co-expression of pptox also led to immunity against K1α, even though this derivative could not interact with the toxin precursor in the secretory pathway. This observation supports the hypothesis of K1 immunity being somehow connected to the secretory pathway by interaction with a so far unknown effector, and is consistent with the defect in immunity observed in many *vps* mutants [4,11]. Likewise, earlier studies have demonstrated that import of α joined to an N-terminal γ fragment into the ER is sufficient to confer immunity [22].

K1 toxin was suggested to act as an ionophore by forming pores in the plasma membrane, either directly after binding to Kre1p or after interaction with so far unknown secondary effectors. Regarding K1 immunity, it was proposed that the K1 protoxin interferes with Kre1p in the Golgi apparatus of the killer yeast, leading to vacuolar degradation of this complex. Both immune and non-immune spheroplasts show normal binding affinity for K1 toxin, indicating that immunity does not rely on the loss of the membrane receptor, but affects a so far unknown step downstream of Kre1p binding [11]. However, so far, a hypothetical participation of Kre1p in intracellular events downstream of the plasma membrane could not be investigated, as the respective null mutant is resistant to externally applied K1 at both the cell wall and membrane level. The α derivatives constructed in this study are able to fill this gap, as they are not dependent on interaction with receptors on the cell wall and plasma membrane. Expression of both α derivatives within a *Δkre1* null mutant yielded a growth defect comparable to the wild type. Likewise, co-expression of K1 pptox yielded functional immunity. These data clearly prove that Kre1p is necessary for neither K1 lethality nor mediation of functional immunity. 

In conclusion, we demonstrate that the K1α derivatives constructed in this study can be successfully used as a novel means to screen for yeast mutants with impact on K1 toxicity and/or immunity. This novel approach could represent a valuable tool for elucidating the underlying molecular mechanisms in future experiments.

## 4. Materials and Methods

### 4.1. Strains, Plasmids, and DNA Manipulation

*E. coli* strain DH5α (F^−^ recA1 endA1 gyr A96 thi hsdR17 supE44 relA1 Δ(argF-lac-ZYA) U169 (Φ80 dlacZΔM15) λ^−^) was used for cloning and amplification of all constructs. Cells were grown at 37 °C in LB medium (1% tryptone, 0.5% yeast extract, and 0.5% sodium chloride) supplemented with 100 μg/mL of ampicillin when necessary. K1 derivatives were generated by PCR using high-fidelity TaqDNA-polymerase (Roche) using primers 5′-K1 (5′-CTC GAG GAA TTC CAT ATG ACG AAG CCA ACC CAA GTA TTA GTT AGA TCC-3′), 3′-K1 (5′-GGA TCC GTC GAC AAG CTT CTA GTG GCC TGT GTC ACA GCC TTC AAA G-3′), 5′-K1alpha (5′-CTC GAG GAA TTC CAT ATG GAA GCG CCG TGG TAT GAC AAG ATC TG-3′), and 3′-K1alpha (5′-AGA TCT GTC GAC AAG CTT TTA AGC AAC GGT AGC GCC ATT AGG ATC TG-3′). After PCR amplification, the corresponding DNA fragments were cloned into pYES2.1/V5-HIS-TOPO (inducible *GAL1* promotor, Invitrogen) according to manufacturer’s instructions and routinely sequenced. Additionally, K1-pptox and K1-tox (pptox lacking the prepro-region) constructs were subcloned into yeast expression vector pYX242 (constitutive *TPI1* promotor, Invitrogen). For in vivo expression of K1 derivatives, *Saccharomyces cerevisiae* strain BY4742 (MATα *his3*Δ*1 leu2*Δ*0 lys2*Δ*0 ura3*Δ*0*) and its corresponding *Δkre1* null mutant were obtained from DharmaconGE (former Open Biosystems). Cells were grown at 30 °C in YPD (1% yeast extract, 2% peptone, 2% glucose). 

### 4.2. Yeast Transformation and Selection 

Transformation was performed by standard lithium acetate method [23], and transformants were grown on uracyl-deficient (ura d/o) synthetic medium supplemented with the appropriate amino acids and nucleotides (0.17% yeast nitrogen base, 0.5% ammonium sulphate, 2% glucose). For co-transformational experiments, media lacked also leucine (ura/leu d/o). Solid media were supplemented with 2% agar. 

### 4.3. Characterization of Suicidal Phenotype

The lethal effect of intracellular expressed K1 derivatives was analyzed by performing serial dilution assays with 5 replicates for each set of experiments. Colonies of the respective transformant were grown in synthetic medium containing 2% glucose and lacking the appropriate amino acids for selection. After incubation for 24 h at 30 °C, cell number was determined, and 10^7^ cells were harvested. Cells were washed twice with synthetic medium containing 2% raffinose to eliminate remaining glucose. Subsequently, logarithmic dilutions ranging from 10^1^ to 10^6^ cells were spotted onto ura d/o or ura/leu d/o (co-transformation) agar plates containing either glucose (uninduced control) or galactose (induced condition); plates were incubated for 3 d at 30 °C.

### 4.4. Flow Cytometry

Potential pore formation was determined by propidium iodide (PI) staining and subsequent flow cytometry. Transformants were grown in galactose-containing ura d/o medium for 24 h at 30 °C to induce expression of the K1 derivatives. Cells were washed twice and transferred into appropriate tubes, followed by addition of PI to a final concentration of 1 µg/mL (stock solution 1 mg/mL in PBS, Sigma). Analysis was performed using a BD LSRFortessa Cell Analyzer and data were analyzed with BD FACSDiva™ software (BD Bioscience, Heidelberg, Germany). Transformants grown under non-induced conditions (glucose) and ethanol-treated cells were used for instrument setting. All samples were measured in triplicate with 50,000 gated events each.
